# Biophysical Approaches for Applying and Measuring Biological Forces

**DOI:** 10.1002/advs.202105254

**Published:** 2021-12-19

**Authors:** Wenxu Sun, Xiang Gao, Hai Lei, Wei Wang, Yi Cao

**Affiliations:** ^1^ School of Sciences Nantong University Nantong 226019 P. R. China; ^2^ Key Laboratory of Intelligent Optical Sensing and Integration National Laboratory of Solid State Microstructure and Department of Physics Collaborative Innovation Center of Advanced Microstructures Nanjing University Nanjing 210023 P. R. China; ^3^ Institute of Brain Science Nanjing University Nanjing 210023 P. R. China; ^4^ MOE Key Laboratory of High Performance Polymer Materials and Technology Department of Polymer Science & Engineering College of Chemistry & Chemical Engineering Nanjing University Nanjing 210023 P. R. China; ^5^ Chemistry and Biomedicine Innovation Center Nanjing University Nanjing 210023 P. R. China

**Keywords:** biological forces, mechanical responses, mechanotransduction

## Abstract

Over the past decades, increasing evidence has indicated that mechanical loads can regulate the morphogenesis, proliferation, migration, and apoptosis of living cells. Investigations of how cells sense mechanical stimuli or the mechanotransduction mechanism is an active field of biomaterials and biophysics. Gaining a further understanding of mechanical regulation and depicting the mechanotransduction network inside cells require advanced experimental techniques and new theories. In this review, the fundamental principles of various experimental approaches that have been developed to characterize various types and magnitudes of forces experienced at the cellular and subcellular levels are summarized. The broad applications of these techniques are introduced with an emphasis on the difficulties in implementing these techniques in special biological systems. The advantages and disadvantages of each technique are discussed, which can guide readers to choose the most suitable technique for their questions. A perspective on future directions in this field is also provided. It is anticipated that technical advancement can be a driving force for the development of mechanobiology.

## Graphical abstract

To gain further insight into how living cells sense and respond to mechanical stimuli, advanced approaches have been developed, including traction force microscopy (TFM), atomic force microscopy (AFM), magnetic tweezer (MT), optical tweezer (OT), molecular force probe (MFP), and micropipette aspiration (MPA). A brief introduction of these techniques can contribute to the investigation of mechanobiology in more detail.

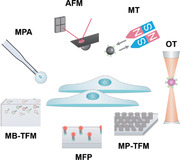

## 1 Introduction

**Figure 1:**
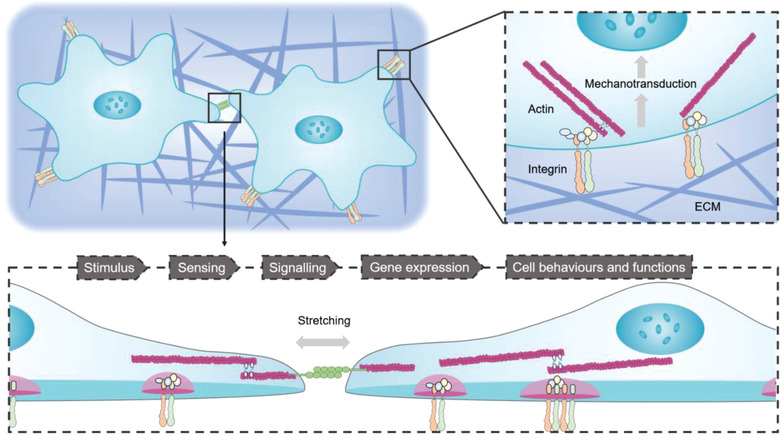
Schematic diagram of the mechanotransduction: cell‐cell (bottom) and cells‐ECM (right). The top left figure depicts cells embedded in the extracellular matrix (ECM). The components of the ECM mainly consist of collagen, fibronectin, and glycosaminoglycans. The top right figure is an expanded view showing the interactions between cells and the ECM and the associated intracellular structures. Typically, cells interact with the ECM mechanically via integrins, which are heterodimeric transmembrane receptors. Mechanotransduction converts mechanical stimuli, such as stretching (through cell‐cell contacts or integrin adhesions), shear stress, and the rigidity of the substrate (through contractile molecules or integrin adhesions), into chemical signals to regulate cell behaviors and functions. The bottom figure shows the pathway of cell‐cell interactions. Mechanical signals can change gene and protein expression profiles by mechanotransduction processes.

Mechanical sensing and response are essential for cells and tissues to retain their normal functions. Stretching, compressive, and shear forces, as well as forces derived from surface tension, are the common mechanical cues that cells are continuously subjected to in vivo. Cells inside tissues are surrounded by other cells and extracellular matrix (ECM). They can communicate by sensing the morphogenesis of neighboring cells,^[^ ^1^ ^]^ sense and respond to the mechanical forces exerted by the ECM^[^ ^2^ ^]^ and then convert the signals to adequate biological responses such as morphogenesis, migration, differentiation, adhesion, and proliferation^[^ ^3^ ^]^ (**Figure** **1**). A recent study has also shown that mechanical cues from the microenvironment around cells, such as substrate rigidity and ECM stiffness, can work together with genetic events to mediate cell fate.^[^ ^4^ ^]^ The epigenetic state and corresponding gene expression that dictate cellular form and function are also regulated by mechanical signals.^[^ ^5^ ^]^ For example, stem cells have drawn great attention in the biological research field owing to their unique differentiation and self‐renewal abilities.^[^ ^6^ ^]^ Their behaviors are closely controlled by mechanical cues from the stem cell niche, a dynamic microenvironment housing stem cells (e.g., ECM and adjacent different cells).^[^ ^7^ ^]^ Appropriately optimizing biomechanical cues is a key factor for obtaining desirable stem cell functions, which can improve the effectiveness of tissue engineering and cell‐based therapy.^[^ ^8^ ^]^ In addition, the maturation and functioning of neuronal cells and the brain, although detailed information is still largely elusive, are strongly regulated by mechanosensing and subsequent mechanotransduction processes.^[^ ^9^ ^]^ Besides, biomechanical cues also play important roles in regulating the processes of alignment and repositioning required in tissue development, homeostasis, and regeneration.^[^ ^10^ ^]^ The interpretation of biophysical intercellular interactions at the level of multicellular systems (e.g., cell sheets and cell spheroids) is fundamentally important to elucidate the substantial correlation between biomechanical stimuli and cell aggregates and tissue formation.^[^ ^11^ ^]^ For the field of bioinspired tissue engineering and biomaterial design, the mechanical properties of culture and regeneration materials can have vital influences on tissues and cells.^[^ ^12^ ^]^ It has been proven that cells can directly sense and respond to the stiffness of surfaces apart from their shape.^[^ ^13^ ^]^ Moreover, emerging evidence has indicated that these cellular mechanosensing processes rely on not only the cell cytoplasm and membrane^[^ ^14^ ^]^ but also cell organelles and nucleus.^[^ ^15^ ^]^ Mechanotransduction processes can be directly or indirectly modulated by the mechanical properties of the nucleus through the physical interaction of the nucleus and cytoskeleton.^[^ ^4^, ^16^ ^]^ Thanks to these findings and understandings, a novel paradigm has been established in which approximating and integrating mechanics must be carefully considered in approaches to engineer biological tissues.^[^ ^17^ ^]^ For example, when considering the formulation and engineering of an artificial 3D support for a cell culture, not only its own biomechanical properties but also cell mechanostimulation and mechanotransduction should be dedicatedly investigated. Additionally, the behaviors of the resident cells can be greatly affected. Abnormal mechanical loading conditions alter cellular functions and ECM properties, eventually leading to tissue pathologies, such as osteoporosis.^[^ ^3^, ^18^ ^]^ Furthermore, these mechanical cues should be considered not only at the bulk level but also at microscale and nanoscale levels in cells, native extracellular matrices, and bioactive molecules. Different cells usually have a characteristic stiffness that is derived from not only their genetic nature but also their communication with the microenvironment. The remarkable review contributed by Reis et al. has minutely introduced and summarized the mechanical properties of different cells and tissues.^[^ ^17^ ^]^ In this review, we simply describe and supplement some basic mechanical characteristics of common cellular components, cells, and tissues (**Table** **1**). As shown in the table and reported by some studies, there are frequently mechanical differences between normal cells and individual cancer cells, which can be used as diagnostic symbols of cancer progression.^[^ ^19^ ^]^ Moreover, evidence has proven that rigidity sensing mechanisms play a vital role in tumor formation and expression patterns of cancer cells.^[^ ^20^ ^]^ As a result, full interpretation of the mechanical changes occurring in cells and tissues can help greatly in some disease diagnoses. Although the mechanical properties were initially considered to be an independent concept, it is now inevitable that they are taken into account in cell and tissue studies.

**Table 1:** Mechanical characteristics of typical biomolecules, extracellular matrix components, different cells, and tissues

Categories			Elastic modulus	Shear modulus	Molecular weight [Mw]	Contour length [nm]	Persistence length [nm]
Biomolecules	DNA	ssDNA^[^ ^221^ ^]^	420–800 pN^[^ ^222^ ^]^		330	0.676	2.223
		dsDNA^[^ ^223^ ^]^	1200 pN^[^ ^224^ ^]^		660	0.34	50
	ssRNA^[^ ^225^ ^]^		150 pN^[^ ^226^ ^]^		320	0.59	1
	F‐action^[^ ^227^ ^]^				42k	4–7	17k
	Polypeptide^[^ ^228^ ^]^				120	0.38	0.4
	Polyethylene glycol^[^ ^229^ ^]^				81	0.41	0.38
	Microtubules^[^ ^230^ ^]^				55k	NA	100–5000k
Extracellular matrix components	Collagen	Molecule^[^ ^231^ ^]^	3–9 GPa				
		Fiber^[^ ^232^ ^]^	100–360 MPa	≈29.4 MPa^[^ ^233^ ^]^			
		Fibril^[^ ^234^ ^]^	2–11.5 GPa	≈33 MPa^[^ ^235^ ^]^			
	Elastin fiber^[^ ^236^ ^]^		1.1–1.2 MPa				
	Fibronectin fiber^[^ ^237^ ^]^		≤3.5 MPa				
	Fibrin fiber^[^ ^238^ ^]^		1–28 MPa				
Cells	MSCs^[^ ^239^ ^]^	Spherical	2.5 kPa				
		Spread	3.2 kPa				
	Osteogenic MSCs^[^ ^240^ ^]^		0.89 kPa				
	Adipogenic MSCs^[^ ^240^ ^]^		0.22 kPa				
	Fibroblasts^[^ ^241^ ^]^		0.5–30 kPa				
	Embryonic stem cells^[^ ^242^ ^]^			≤2 kPa			
	Pancreatic cells^[^ ^19a^ ^]^	Healthy cells	0.54 kPa				
		Carcinoma	0.54 kPa				
	Pleural effusion cells^[^ ^243^ ^]^	Healthy cells	2.53 kPa				
		Metastatic cells	0.38 kPa				
	Thyroid cells^[^ ^244^ ^]^	Healthy cells	1.2 kPa				
		Cancer cells	1.3 kPa				
	Human breast cells^[^ ^245^ ^]^	Normal cells	1.75 kPa				
		Tumorigenic cells	0.3 kPa				
Tissues	Connective tissue	Bone^[^ ^246^ ^]^	10.4–20.7 GPa	3.28–7 GPa			
		Ligament^[^ ^247^ ^]^	25–93 MPa	0.045–1.7 MPa			
		Cartilage^[^ ^248^ ^]^	0.24–0.85 MPa	5.7–6.2 MPa			
	Muscle tissue	Cardiac muscle^[^ ^249^ ^]^	8 kPa	5–50 kPa			
		Skeletal muscle^[^ ^250^ ^]^	5–170 kPa				
	Nervous system	Brain^[^ ^251^ ^]^		1–3 kPa			
		Nerve^[^ ^252^ ^]^	5 MPa				
	Skin^[^ ^253^ ^]^		60–850 kPa	4–12 kPa^[^ ^254^ ^]^			
	Liver^[^ ^255^ ^]^		4.0–6.5 kPa	2 kPa			
	Thyroid^[^ ^256^ ^]^		9–50 kPa	1.3–1.9 kPa			

The key components of mechanotransduction are mechanosensors and motor proteins on actin filaments anchored to cells or the ECM.^[^ ^21^ ^]^ However, how cells sense and respond to mechanical signals is still largely unknown. Mechanobiology is a multidisciplinary field that concerns the underlying mechanisms of mechanical sensing, transduction, and response in cells^[^ ^22^ ^]^ (Figure 1). Mechanobiological studies at the cellular level require tools that can 1) exert mechanical loads on cells and 2) quantify cellular forces and the shape changes in cells. To achieve these goals, novel measurements using microbead‐based traction force microscopy (TFM), micropillar‐based TFM, atomic force microscopy (AFM), magnetic tweezers (MTs), optical tweezers (OTs), molecular force probes (MFPs), and micropipette aspiration (MPA) have been developed. These platforms allow researchers to quantitatively analyze the mechanical properties of the cell microenvironment and characterize the downstream mechanical responses of cells, such as rigidity sensing, shape change, and expression of proteins.^[^ ^22^, ^23^ ^]^ These studies provide insights into the functions of different components and the network involved in these mechanotransduction processes. In the following sections, we will discuss the development of these techniques and their applications in mechanobiology.

## 2 Microbead‐Based TFM

**Figure 2:**
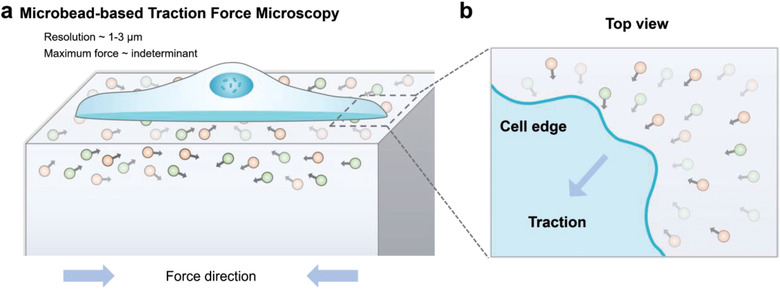
a) Schematic diagram of microbead‐based traction force microscopy. b) Top view of the schematic diagram of the traction generated on the cell edge. Cell type, substrate properties, and cell shape can greatly influence the generated force. Fluorescent beads were embedded in elastic gels, and the movements of the beads were tracked. Fluorescent beads are often of two different colors to improve the spatial resolution. The dynamic cellular forces on continuous surfaces can be calculated.^[^ ^28^ ^]^

Cells need to adhere to solid substrates to survive and grow. When adhered to surfaces, cells develop traction forces to organize ECMs, maintain cell shape, probe physical environments, and generate mechanical signals.^[^ ^24^ ^]^ An approach for measuring cell traction forces is urgent for a better understanding of the underlying mechanisms of mechanosensing. The total traction force generated by single cells depends on cell type, substrate properties, and cell shape. Several tools have been developed to determine the traction force^[^ ^25^ ^]^ (**Figure** **2**). Typically, the traction force transmitted to substrates through focal adhesions and individual focal adhesion can generate forces of ≈10 nN.^[^ ^26^ ^]^ The first experiment to detect the force generated by cells proceeded by using an elastic silicone surface as the substrate. The cells wrinkle the elastic silicone rubber substrate during locomotion, which makes the force “visible.” This experiment showed that non‐muscle cells generated force in their environment.^[^ ^27^ ^]^ However, this method cannot track the locomotion of cells because of its low spatial resolution. Moreover, due to the nonlinear deformation of silicone rubber, it is difficult to measure the force precisely. Then, methods of embedding markers in elastic substrates, such as polyacrylamide gels, have been developed. By doing so, wrinkling can be avoided, and the displacement of markers can be observed by optical microscopy, which directly indicates the local deformation exactly. Finally, the traction force and cell shape change can be reconstructed by using elastic theory and finite element analysis.

As the most widely used substrates for traction force measurements, polyacrylamide gels are transparent and can be combined with fluorescence microscopy to analyze the fluorescence‐labeled cytoskeleton or focal adhesion.^[^ ^29^ ^]^ The mechanical properties of the gels are determined by the ratio of acrylamide and bis‐acrylamide and the acrylamide concentration. The stiffness can be modified over a wide range from 1.2 to 100 kPa^[^ ^30^ ^]^ to mimic the rigidity of different tissues.^[^ ^31^ ^]^ To improve the spatial resolution and accuracy of the measurement, many studies have been undertaken. Among them, the use of substrates labeled with two or more different fluorophores at a relatively high density shows high spatial resolution (Figure 2).^[^ ^32^ ^]^ A key factor affecting the spatial resolution of microbead‐based TFM is the quality of the reconstructed traction field. The images of fluorophores at different colors can be acquired in different channels of the multispectral confocal microscope. Thus, high information contents including displacement field are achieved subsequent by cross‐correlation calculation. With the help of these materials, it is possible to characterize the dynamics and distribution of traction forces with specific focal adhesions.^[^ ^2^ ^]^

To characterize the cell traction force, a reconstruction process is needed that transforms the given displacement field to the traction force field. The reconstruction process is based on the theory of linear elastostatics.^[^ ^33^ ^]^ The displacement at point *x_j_* of an elastic substrate due to *n* cell traction forces can be written as:^[^ ^32^, ^33^ ^]^

(1) $$d\;\left(x_{j}\right)=\sum_{i=1}^{n}G\left(x_{i}-x_{j}\right)\cdot F\left(x_{i}\right)$$

where *F*(*x_i_*) is the force exerted at *x_i_*, and *G*(*x_i_* − *x_j_*) is Green's function depending on the gel properties and the boundary conditions. The earliest studies have used the Boussinesq solution,^[^ ^33^ ^]^ which gives the surface displacement of an infinite half‐space due to a point surface load.^[^ ^30^, ^34^ ^]^ However, the Boussinesq solution overestimates the displacement due to the finite thickness of gel substrates. By deconvolving this equation, the forces can be readily found. The construction of matrices and the calculation of the equation may require high computational costs. If the sites of adhesion can be determined by fluorescent labels, then the force can be localized to a smaller area. It will be more effective to reconstruct the traction of point forces.^[^ ^35^ ^]^

Since the result depends on the thickness and shape of the gel, the finite element method (FEM) can provide a more accurate estimation of the traction forces.^[^ ^36^ ^]^ The FEM allows us to reconstruct the traction of a 3D system with arbitrary shape and local properties. For example, FEM has been used to calculate the traction force of cells encapsulated in PEG hydrogels.^[^ ^37^ ^]^

To solve the inversion problem, both methods are computationally demanding. In addition, the uneven distribution of the fiducial markers would lead to irregular resolution of the force field. To solve this problem, the microcontact printing method has been used to create regular arrays of embedded markers.

To increase the spatial resolution of conventional microbead‐based TFM and improve measurement accuracy, many studies have been devoted to devising and engineering superresolution TFM. The technical approaches can be divided into two different branches. First, technique optimization is a straightforward but useful method. Additionally, with the development of high‐resolution imaging techniques, the precision of TFM has been greatly enhanced, and this approach has been widely used in a variety of fields in biological research.^[^ ^32^, ^34^, ^38^ ^]^ For example, Hakanpaa et al. investigated the importance of *β*1‐integrin for the inhibition of vascular leakage in endotoxemia through confocal‐based analyses.^[^ ^38c^ ^]^ The mechanical stress exerted by endothelial cells (ECs) was measured. Their results showed that the contractility of thrombin‐induced endothelial cells was decreased by mAb13, which led to the dissolution of VE‐cadherin from EC junctions. Moreover, their simulation and calculation also indicated that *β*1‐integrin is an active promoter in vascular leakage. As a result, a novel means to stabilize vasculature in vascular leaks can be achieved by targeting the *β*1‐integrin signaling pathway. In addition, the combination of deformable hydrogel microparticles and TFM has recently shown great promise for broad biological and biomedical applications.^[^ ^39^ ^]^ To precisely assess the cellular interactions on a 3D level, Vorselen et al. developed a particle‐based force‐sensing scenario.^[^ ^39a^ ^]^ Traditional fluorescent beads were replaced by deformable and tunable hydrogel particles, which possibly mimic the rigidity, size, and chemical characteristics of living cells. Equipped by such micro hydrogel particles, these researchers successfully investigated force dynamics in T‐cell immunological synapses and the subcellular force distribution throughout phagocytic engulfment.

On the other hand, the reconstruction algorithm is a critical factor that affects the sensitivity and accuracy of TFM.^[^ ^40^ ^]^ The computational algorithm devised by Han et al. successfully identified cellular tractions in diffraction‐limited nascent adhesions.^[^ ^41^ ^]^ However, requiring heavy computational processing power is the main shortcoming that limits its applications. Recently, a simplified imaging strategy named the fluctuation‐based super‐resolution (FBSR) algorithm developed by Stubb et al. has skillfully enhanced the output of microbead‐based TFM by increasing not only the trackable bead density but also the tracking accuracy.^[^ ^42^ ^]^ The light intensity fluctuation of the fluorophores caused by the transitions between nonfluorescent and fluorescent states contributes to the improved resolution in the prediction of the location of fluorophores. In this way, they investigated the filopodia alignment along the force field generated by focal adhesions. There is no doubt that novel improvement of experimental and reconstruction methods in TFM can open doors in mechanotransduction studies.

## 3 Micropillar‐Based TFM

**Figure 3:**
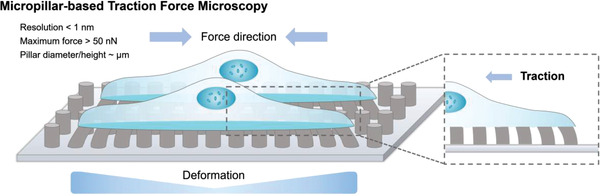
Schematic diagram of micropillar‐based traction force microscopy. The stiffness of the micropillars is dependent on their material and dimensions (diameter and height). When the cells migrate or respond to mechanical stimuli, the associated micropillar will bend and change its shape. The forces can be calculated based on these changes.^[^ ^43a^ ^]^

As an alternative strategy to microbead‐based TFM using flat and continuous substrates, a microfabricated post array or micropillar array enables the measurement of cell‐derived forces by observing pillar bending.^[^ ^43^ ^]^ This technique uses a substrate with evenly spaced micron‐sized pillars to sense cell locomotion (**Figure** **3**). For a small deformation or bend, the pillar behaves as a Hookean spring, and the force *F* is proportional to the deformation *δ*:

(2) F=kδ

where *k* is the spring constant. According to the bending formula, the spring constant can be written as:

(3) $$k=\frac{3\pi ER^{4}}{4L^{3}}$$

where *R*, *L* and *E* are the pillar radius, pillar height, and Young's modulus, respectively.

Because each pillar moves independently, this technique avoids heavy computational overhead and directly measures local traction by the bending of individual pillars. The rigidity of pillars can be controlled by the aspect ratio and width of the pillar instead of the materials. Therefore, the substrate rigidity can be rapidly tuned to study the corresponding cell responses. The micropillar array technique has been applied in studies of fibroblasts,^[^ ^43^, ^44^ ^]^ stem cells,^[^ ^18b^ ^]^ epithelial cells,^[^ ^45^ ^]^ endothelial cells,^[^ ^46^ ^]^ neutrophils,^[^ ^47^ ^]^ T‐cells,^[^ ^48^ ^]^ and platelets.^[^ ^49^ ^]^

Additionally, the top of the pillars can be chemically modified with covalently linked ligands that bind to specific cell surface receptors, such as integrin *α*5*β*1, and quantify the contribution of the ligands to the traction forces.^[^ ^50^ ^]^ However, this technique raises some concerns that the gaps between pillars may change the cell geometry and traction force patterns.^[^ ^43a^ ^]^ Micropillar array substrates have been combined with different techniques to study different problems. For example, it can be incorporated into microfluidic devices to investigate the effect of shear flows on cells.^[^ ^51^ ^]^ Also, it can be used on top of a stretchable substrate to study mechanosensing in smooth muscle cells.^[^ ^52^ ^]^

Traditional micropillar array substrates are fabricated with negative molds. In the earliest approach, photolithography is used to pattern “pit” arrays on silicon wafers, and the wafers are used as negative molds. Then, the silicon mold is salinized to prevent sticking of the substrate. A layer of polydimethylsiloxane (PDMS) is spin‐coated onto the mold and cured. In the last step, the PDMS layer is peeled and functionalized by protein ligands or fluorescence labels. Functionalization can be performed by microcontact printing, including by PDMS molds and liquid drops through the “lotus effect.”^[^ ^43^, ^53^ ^]^ Another way to prepare a micropillar array is to use nanofabricated pillars directly on the silicon wafer, and a negative PDMS mold is then created by the silicon mold. The micropillar array is prepared by using a negative PDMS mold. The first step for the fabrication of silicon molds is to spin‐coat the photoresist on a clean silicon wafer. Then, the photoresist is exposed to ultraviolet (UV) light passing through a photomask, and the pattern of the photomask will determine the size and spacing of the pits. The photoresist is then developed, and plasma etching is used to generate pits at the desired depth. The selectivity of the photoresist is usually the limitation for making a high‐aspect‐ratio pillar. To overcome this disadvantage, an additional temporary chromium mask is coated on silicon. The Cr mask has a much higher resistance to plasma etching and can greatly increase the aspect ratio.^[^ ^43b^ ^]^

To be used as force sensors, the mechanical properties of the fabricated substrates must be characterized. The stiffness of the micropillars is dependent on their dimensions (diameter and height) and material. The dimensions can be measured from scanning electron microscopy images. The mechanical properties of PDMS, such as Young's modulus and Poisson's ratio, can be measured from bulk experiments,^[^ ^54^ ^]^ beam theory, or FEM calculations. In addition, a delicate approach based on AFM with contact model imaging (CMI) and force spectroscopy imaging (FSI) modes was devised by Angeloni et al. to directly determine the mechanical characteristics of the macro‐ and nanopillars.^[^ ^55^ ^]^ The global adaptation, detection, and response behaviors of cells to the rigidity of matrices have recently been studied systematically by micropillar array‐based approaches.^[^ ^56^ ^]^

Derived from the design strategy of micropillar‐based TFM, technical approaches using microstrips, micropatterns, and functionalized micropillars have been exploited as effective tools to investigate and manipulate cell behaviors.^[^ ^8^, ^57^ ^]^ Via electron beam lithography (EBL), Dalby et al. successfully detected and determined how cells will respond to nanoscale landscapes.^[^ ^58^ ^]^ The nanopatterns used to culture and track cells consist of a batch of pits with hexagonal, square, displaced square, and random placements. Their results demonstrated the possibility of employing nanoscale disorder to stimulate hMSCs to produce bone minerals in vitro in the absence of osteogenesis, which endows topographical treatment with promising candidates for cell therapies. Similarly, Mohammed et al. investigated the influence of substrate spatial confinements on collective cell migration by adhesive microstrips.^[^ ^59^ ^]^ The direct correlation between the cell‐substrate adhesive area and the velocity of the confined cells was elucidated through their findings. To further expand the applications of micropillar‐based approaches, Hansel et al. developed mesoporous silicon nanoneedle arrays that can interact simultaneously with different cellular components of primary human cells, including the cell membrane, cytoskeleton, and nucleus.^[^ ^60^ ^]^ Their results revealed that such nanoneedles can reduce tension in the cytoskeleton, inhibit focal adhesion maturation at the membrane, and lead to remodeling of the nuclear envelope at sites of impingement. Herein, these authors finally highlight the ability of nanoneedle arrays to guide the phenotype and behaviors of large cell populations simultaneously by regulating the mechanotransduction processes of the cells. As a new platform to manipulate cells in vitro, Amy Sutton and coworkers developed a new type of active substrate for cell culture.^[^ ^61^ ^]^ A polymeric array of microstructure actuators is embedded in a stimuli‐responsive hydrogel layer, and the microstructure tips can serve as the focal adhesion point for cells. When a laser beam is focused on a point of the hybrid substrate, the hydrogel will contract, and the microstructure will subsequently bend. As a result, the anchorage points on the microstructure tips for focal adhesion will be displaced by several microns. Owing to the compatibility to deform the cell growth surface in a highly controlled manner, this new cell culture platform becomes a promising candidate to study how mechanical signals propagate inside single cells and populations of cells. The local curvatures on the cell membrane can also serve as a kind of mechanical signal to modulate a batch of cellular processes.^[^ ^62^ ^]^ To investigate the influence of local curvatures more directly, Martino et al. engineered a cell culture surface consisting of light‐responsive polymer nanostructures whose shape can be dynamically tuned by light.^[^ ^63^ ^]^ Upon green light illumination, the azobenzene‐based polymer nanostructures used in this material can change from vertical pillars to elongated vertical bars. Their results elucidated that the high membrane curvatures at bar ends induced by such reshaping processes promote the local accumulation of the actin nucleator Arp2/3 complex and actin fibers. The ability to control the curvatures of this platform on demand precisely opens up a new way to study curvature‐dependent processes in live cells. Altogether, these studies highlight the great potential of micropillar‐based TFM methods to manipulate cell behaviors by regulating the traction forces across a hierarchy of scales.

## 4 AFM

**Figure 4:**
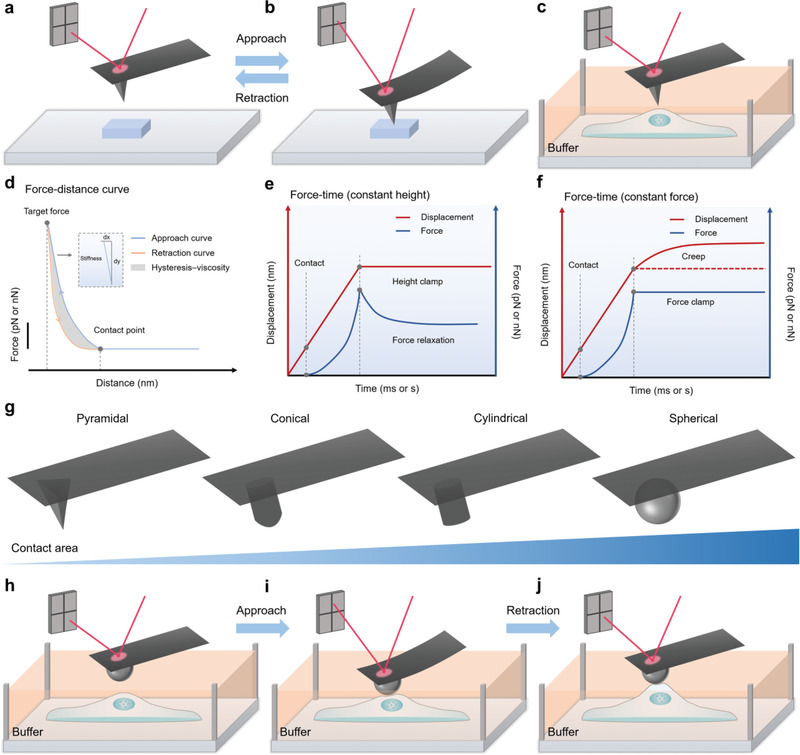
Schematic diagram of the atomic force microscopy (AFM)‐based investigation approaches. a) Schematic illustration of the basic setup of AFM. A laser beam is reflected by the cantilever and detected by a position‐sensitive detector (PSD). b) When the probe contacts the sample, the cantilever bends. The position of the reflected laser on the PSD changes accordingly. c) For biological samples, AFM measurements can be performed in a fluid chamber. The buffer composition and environmental conditions can be precisely controlled. d) An example of a force‐distance (FD) curve. The blue curve is the approach process, and the red curve is the retraction process. Based on the FD curve, the contact position between the probe and the sample can be determined, and the stiffness of the sample can be estimated. e) An example of a force‐time (FT) curve (constant height). The probe indents the sample and is kept at a constant height. The recorded force can be used to analyze the mechanical response of the sample. f) An example of a force‐time (FT) curve (constant force). The probe indents the sample, and the cantilever is kept at a constant deflection. The recorded displacement of the cantilever can be used to analyze the mechanical response of the sample. g) A series of probes can be used for the measurements of various biological systems. The contact area between the probe and biological system depends on the shape and geometry of the probe. The larger the contact area is, the more suitable the probe is to measure the average properties over a larger sample area. h) Spherical probes are better than common pyramidal probes for the measurement of a soft and dynamic biological system. i) Approaching process of a spherical probe during the measurement. j) Retraction process of a spherical probe during the measurement.

AFM has been used in a wide variety of fields in mechanobiology. In an atomic force microscope, a cantilever, which is several to hundreds of micrometers in length and has a specified probe at the end, is used to interact with the sample and detect the forces between the probe and the sample with piconewton sensitivity (**Figure** **4**a). The movement of the cantilever is controlled by a piezoelectric positioner. When the probe is pressed or stretched, the cantilever bends, and the bending angle is proportional to the force acting on the probe according to Hooke's law (Figure 4b). A laser beam is reflected by the cantilever and detected by a position‐sensitive detector (PSD). After calibration, the force can be mapped out from the detector signal, and the deformation can be obtained by considering both the movement of the piezoelectric positioner and the bending of the cantilever. Depending on the stiffness of the cantilevers, a wide range of samples, from tissues to single molecules, can be studied by AFM.^[^ ^3^, ^64^ ^]^ Establishing suitable experimental conditions is critical for the success of the measurements.

To investigate the mechanical properties and mechanotransduction of living cells, maintaining physiological conditions during measurement is essential. Therefore, both the cantilever and samples are typically immersed in a fluid chamber filled with buffers. With the help of an environmental controller system, the temperature and other conditions can also be controlled precisely. As a result, AFM allows characterization under predefined conditions, such as a suitable pH value, temperature, humidity, CO_2_ concentration, and buffer composition (Figure 4c). At the single‐molecule level, AFM has been used to measure the mechanical response of many mechanical proteins, for example, revealing the unfolding of individual immunoglobulin and fibronectin‐like domains in muscle protein titin. The unfolded domains can refold after the force is relaxed, suggesting that the muscle has a mechanism to stabilize itself during overstretching.^[^ ^64^, ^65^ ^]^ AFM has also been used to study various ligand‐receptor interactions.^[^ ^66^ ^]^ Furthermore, subcellular mechanics play a vital role in virus‐cell interactions, which can affect the ability of viruses to enter cells.^[^ ^67^ ^]^ Mechanical measurements of human immunodeficiency virus (HIV) particles using nanoindentation technologies based on AFM revealed a maturation‐induced “stiffness switch” mechanism in its life.^[^ ^68^ ^]^

A great challenge in cellular mechanobiology is to exert precise mechanical loads on cells and measure their feedback simultaneously. In a typical AFM experiment, a probe indents the cell surfaces until a defined force is reached and then retracts to a preset position. By detecting the deflection of the cantilever, the force‐distance (FD) curves can be quantitated (Figure 4d). Based on the FD curves, the mechanical properties of the cells of interest can be analyzed. The most basic theoretical model for quantifying mechanical parameters from AFM‐based FD curves is the Hertz model. It assumes that the sample touched by the probe is purely elastic, expands infinitely, and shows no substructures.^[^ ^23c^ ^]^ These general assumptions make the model of limitations in practice. Most biological samples only show pure elasticity at strains less than 20%. In addition, the probe should be blunt. To make the elastic stress and strain depend linearly on Young's modulus E (*σ*  =  E*ε*), the indentation depth needs to be less than 10% of the sample thickness, and the contact area between the probe and the sample should be much smaller than the sample dimensions.^[^ ^69^ ^]^ Moreover, the deformation of the samples should be fully reversible. However, most complex biological systems, such as cells and tissues, usually show viscoelastic behaviors. Between the approach and retraction FD curves, there is always hysteresis (Figure 4d). As a result, the viscosity *η* should be considered in the stress‐strain relationship. Although the Hertz model is suitable and effective in most cases, it does not take into account surface forces, such as the adhesion and friction between the probe and samples. However, friction and adhesion are usually inevitable when the probe is in contact with the biosystems.^[^ ^70^ ^]^ To minimize this effect, modifying the probe surface with nonadhesive polymers, such as polyethylene glycol, has been proven to be an effective method.^[^ ^71^ ^]^ Alternatively, two other models developed from the Hertz model are suitable for situations with surface forces, including the Johnson–Kendall–Roberts model^[^ ^72^ ^]^ and Derjaguin–Müller–Toporov model.^[^ ^73^ ^]^ These two models were originally used to analyze the interaction between spherical probes and flat surfaces. Moreover, after slight modification, they can be extended to probes with other shapes and geometries, such as a conical probe.^[^ ^74^ ^]^ Additionally, the soft substrate also affects the accurate measurement of the rigidity of the cells. Studies have shown that the deformation on the underlying substrate is unignorable if the rigidity of the substrate is lower than that of the cells.^[^ ^75^ ^]^ To overcome this obstacle, Franze et al. developed a “composite cell‐substrate model” that made a significant contribution to the understanding of many physiological and pathological processes.^[^ ^75^ ^]^ Besides the FD curves, force‐time (FT) curves can be plotted, in which the force is plotted against time^[^ ^76^ ^]^ (Figure 4e,f). When the depth of the probe or the target force is controlled to be constant, these FT curves are particularly useful. Because live cells constantly change their mechanical properties and show a time‐dependent mechanical response, the FT curves can better detect and analyze cell behaviors in these cases^[^ ^77^ ^]^ (Figure 4e,f). The Hertz model is also suitable for extracting relative mechanical parameters from FT curves.^[^ ^23c^ ^]^

Even if the experimental process fully meets the assumption of adapting the Hertz model, whether the experimental parameters and conditions are carefully controlled is a critical factor for accurate measurements. A critical factor to measure the mechanical properties of a biological system of interest precisely is to select cantilevers with spring constants similar to those of the biosystem.^[^ ^23c^ ^]^ If the cantilever is much softer than the biological system, then the deflection becomes insufficient to estimate the sample stiffness. In contrast, if the spring constant of the cantilever is much higher than that of the system, the measurement becomes insensitive because the deflection of the laser on the PSD becomes minimal. In addition to the spring constant, there are a series of different types of probes that can be selected. AFM probes often have well‐defined shapes, and the dimensions range from the micrometer to nanometer scale (Figure 4g). The main difference between these probes is the contact area. A probe with a larger contact area is more suitable for measuring the average properties over a larger sample area. To measure the mechanotransduction of living cells, spherical probes may be more suitable than common pyramidal probes.^[^ ^78^ ^]^ As the probe continues going deep into the cell, it becomes increasingly difficult to estimate how it interacts and deforms the cells (Figure 4h,j). Moreover, the spherical probe can describe the mechanical properties of a heterogeneous sample more precisely. After an accurate measurement, analyzing the resulting curves correctly is important for extracting the mechanical properties. Defining the contact point between the probes and the samples is the first step in the analysis. However, most living cells or other biosystems have complex matrix structures and surface morphologies. The contact point on the FD curves may be unclear, causing an inaccurate determination of the indentation depth. To avoid this inaccuracy, the preset indentation needs to be at least 400 nm.^[^ ^79^ ^]^ The loading rate adapted during measurements can greatly affect the mechanical properties of most biosystems. The internal components of biosystems are dramatically complex, and the responses of various components to the loading rate are quite different. As a result, the mechanical properties of biosystems usually change nonlinearly with the loading rate.^[^ ^80^ ^]^ Therefore, it is meaningless to compare the mechanical properties of different biosystems without the same loading rate. Additionally, materials with complex components and structures often respond differently to different mechanical cues, such as tension, indentation, shear, and friction. Thus, designing AFM experiments carefully and analyzing the data accurately is the key to drawing correct conclusions.

How native biosystems sense, transduce, and respond to mechanical cues are fundamental challenges in mechanobiology. AFM‐based nanoindentation approaches can achieve measurements in various biomolecular systems and quantify their mechanical properties precisely, including deformation, tension, compression, friction, and energy dissipation. Structured networks that consist of semiflexible actin filaments play an important role in regulating cell stiffness and transmitting forces during mechanotransduction and cell motility.^[^ ^81^ ^]^ A reversible stress‐softening regime of dendritic actin networks while the stresses increased above a critical point (270 Pa) was observed by Chaudhuri et al. using a modified AFM to apply forces sinusoidally.^[^ ^82^ ^]^ This reversible stress‐softening regime, followed by stress‐stiffening behaviors, is considered to be related to the self‐protection mechanism of the network under compression. Furthermore, much attention has been given to the regulatory effect of dynamic molecular processes on cellular mechanotransduction,^[^ ^3a^ ^]^ and more detailed information on various dynamic processes has been gathered by AFM‐based nanoindentation approaches.^[^ ^83^ ^]^ Also, AFM has been employed as a passive force monitor in the study of cell migration to directly probe the biological forces generated by lamellipodial protrusion.^[^ ^84^ ^]^ The resulting force curves indicated the complex multiphase processes of protrusive force generation, relating to action and adhesion dynamics. Besides the dynamic molecular measurements, nanoindentation approaches based on multi‐harmonic AFM can achieve local mapping of different mechanical properties, including local stiffness, stiffness gradient, and viscoelastic dissipation of live cells with relatively high throughput and resolution.^[^ ^85^ ^]^ These mechano‐mapping technologies with outstanding efficiency and precision have been applied to analyze mechanical changes in tumors and have been further developed to be a tool for diagnosing cancer.^[^ ^86^ ^]^

However, one main drawback of AFM‐based approaches is that mechanical stimuli can only be applied outside of the biosystem. To better characterize the mechanotransduction and response of a cellular system, some specific technologies have been introduced to combine with AFM.^[^ ^87^ ^]^ For example, during AFM measurements, nanomaterials or micromaterials can be used to mechanically stimulate or confine the cellular system from another direction.^[^ ^23c^ ^]^ Microfluidic devices, nanopillars or micropillars, and elastic substrates are common supporting approaches.^[^ ^88^ ^]^ Moreover, fluorescent labeling approaches have also been used together with AFM. Fluorescent molecules can label specific structures inside cells or biosystems. Additionally, the 3D piezo stage can provide tunable motion of the cantilever, such as shearing. Although AFM is mainly used to investigate the local mechanical distribution on the cell surface, recently, some modified AFMs equipped with special probes have been successfully used to study the mechanical properties of intracellular nuclei and organelles.^[^ ^89^ ^]^

Currently, AFM‐based multifunctional toolboxes have been considered one of the most promising research strategies in mechanobiology.^[^ ^23^, ^90^ ^]^ AFM‐based nanoindentation approaches can exert force actively and precisely on local areas of cells and tissues. Using this advantage, Elosegui‐Artola et al. revealed force‐driven YAP nuclear translocation mechanisms.^[^ ^91^ ^]^ A constant force can be directly applied to cell nuclei by AFM. Such mechanical forces further modulate the structure of nuclear pores and hence regulate the nuclear translocation of YAP. To assess the importance of mechanical cues for axon growth in vivo, Koser et al. employed in vivo AFM (iAFM) together with a series of biological manipulations, including knocking down the mechanosensitive ion channel piezo1 and pharmacologically blocking mechanotransduction.^[^ ^92^ ^]^ iAFM technology was developed to map the local mechanical properties of the exposed intact developing brain at different developmental stages by force‐indentation tests. Their results demonstrated that all treatments led to pathfinding errors and aberrant axonal growth, indicating that the local mechanical properties of the surrounding tissues sensed by mechanosensitive ion channels are critical for axonal growth patterns in vivo. Similarly, benefiting from iAFM technology, a recent study by Elias H. Barriga and coworkers successfully characterized mechanical changes obtained during neural crest migration in heterochronic tissue grafts.^[^ ^93^ ^]^ Moreover, by performing mechanical and molecular manipulation, these researchers finally concluded that altering tissue stiffness is a key factor in triggering collective cell migration because of the promotion of the epithelial‐to‐mesenchymal transition in vivo. AFM has also been equipped as a tool to actively confine cells on a cellular scale for the investigation of cell movement and migration.^[^ ^94^ ^]^ Lomakin et al. combined AFM‐based dynamic confinement, force measurements, and live cell imaging to quantitively assess cell responses to their mechanoenvironment.^[^ ^94^ ^]^ A setup containing an AFM with an ion beam‐sculpted flat silicon microcantilever was used to confine single cells. Simultaneously, confocal video microscopy and AFM‐based force spectroscopy were introduced to monitor and calculate the contractile forces and myosin cytoskeleton dynamics. The results revealed that immune and cancer cells can sense confinement through the deformation of their nucleus. The bounding nuclear envelope (NE) starts to unfold and stretch once the compression exceeds the size of the nucleus. Subsequently, the tension increase in the nuclear membrane will trigger calcium release and activate the enzyme cPLA2, which initiates an “evasion reflex” mechanism to help the cell escape rapidly out of its compressive microenvironment. This mechanism was also confirmed by Venturini et al.^[^ ^95^ ^]^ The nonlinear viscoelastic properties of living cells are closely related to cell morphology and state. Because microcantilevers can possess relatively high resonance frequencies, AFM‐based technologies have achieved the rheological characterization of living cells at higher frequencies, which is difficult to accomplish by traditional passive and active rheology techniques.^[^ ^79^, ^96^ ^]^ The setup developed by Fläschner et al. consists of two parallel microcantilevers (master and slave microcantilevers), and a round cell is confined between the two microcantilevers.^[^ ^97^ ^]^ In a typical measurement, the master microcantilever is driven by a blue laser under a desired frequency, and the motion of the slave microcantilever is read and recorded by a red laser, hence quantifying the cell viscoelastic properties. Together with optical microscopy and cell mass measurement, their results revealed that there is no correlation between cell size and viscoelasticity, which defies an assumption based on Laplace's law. In addition to the development of AFM instruments, a computational method devised by Garcia et al. can directly transform the experimental data from the AFM‐based FD curves into viscoelastic parameters of the living cells as a function of frequency.^[^ ^98^ ^]^

Therefore, AFM‐based approaches have both advantages and limitations for the investigation of mechanotransduction. The design of AFM allows measurements of a wide variety of mechanical properties of living systems, and the mechanical cues applied by AFM can be well defined and carefully devised. The forces applied and measured by AFM can range from piconewtons to micronewtons. Besides, the spatial areas mapped by this method range from subnanometers to several tens of micrometers. Recently, with the help of the developed algorithm, AFM can perform mechanical property mapping on a defined area in the time range from hours to milliseconds (mainly depending on the size of the area). AFM‐based approaches with greatly enhanced throughput have been successfully employed to simultaneously investigate the morphological and nanomechanical properties of several hundred extracellular vesicles on the timescale of hours.^[^ ^99^ ^]^

## 5 Magnetic‐Nanomaterial‐Based Approaches

**Figure 5:**
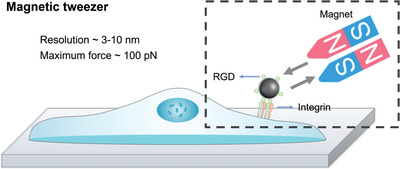
Schematic diagram of the magnetic‐nanomaterial‐based approaches. Magnetic tweezers create a magnetic field by electromagnets or permanent magnets. The magnetic field can control magnetic nanomaterials to apply forces to target molecules in vitro or in vivo, such as integrins with RGD‐modified beads.^[^ ^107^ ^]^

In contrast to AFM‐based mechanotransduction investigation methods, magnetic nanomaterial (MN)‐based technologies can remotely study and control cell behaviors^[^ ^100^ ^]^ (**Figure** **5**). The forces exerted on cells are generated by a magnetic field that has adjustable field strength and low attenuation in biological organisms. This approach provides an external trigger for cell fate regulation. The forces for manipulation of mechanically sensitive biomolecules are reported to be in the range of piconewtons (pNs).^[^ ^22^, ^101^ ^]^ For example, the mechanotransduction channels in hair cells can be opened by a single magnetic force pulse at ≈0.29 pN.^[^ ^102^ ^]^ This value falls into the range that magnetic‐based approaches can generate correctly.^[^ ^22^, ^103^ ^]^

The mechanical stimuli applied to the biosystems are generated through magnetic beads whose motions are controlled by a magnetic field. The magnetic fields can be divided into two types: static and dynamic fields. It has been proven that both types can greatly affect mechanotransduction. Static magnetic fields are often produced by permanent magnets. The field gradient^[^ ^104^ ^]^ can reach the range from 1 to 100 T m^−1^. Electromagnets and movable permanent magnets are usually applied to generate dynamic magnetic fields.^[^ ^105^ ^]^ Among the two devices, electromagnets are more conveniently controlled and allow the production of time‐dependent force fields. To produce a magnetic field with a strong gradient, a single‐pole electromagnet with a sharp tip is introduced into the devices. Under such a configuration, the magnetic field gradients near the tip region are tremendously large. The forces on the magnetic beads can be described as a function of the distance between the tip of the electromagnet and the bead. Note that the electromagnets are usually adapted in pairs, which can produce a constant magnetic field gradient to obtain a relatively homogeneous force field over a wide area. Besides, to control the rotations and multiple directions of the magnetic beads at the same time, multiple pairs of electromagnets can be used.^[^ ^106^ ^]^

The composition and structure of the MN play a key role in contributing to magnetic properties. Element selection, cation distribution control, and doping are the common methods for composition optimization to improve the magnetic properties of MNs. In addition, a positive correlation is the typical relationship between the saturation magnetization of MNs and their size.^[^ ^108^ ^]^ When the size is small enough, the orientation of the magnetization can be easily affected by thermal fluctuations.^[^ ^109^ ^]^ Moreover, employing ions such as Co, Zn, and Mn can further enhance the magnetization of MNs.^[^ ^23^, ^110^ ^]^ The function of the ions is to either reduce off‐site magnetic spins or add more unpaired electrons. The unique feature of magnetic ferrite nanoparticles (MFNPs) is that they can produce local magnetic fields by responding to an external magnetic field, making them the most attractive candidates for biomedical applications.^[^ ^111^ ^]^ Besides, a specific shape can also optimize the net magnetic magnetization. For example, compared to spherical MNs, cubic MNs exhibit better magnetization properties at the same size. The reason is that the disordered spins are broadly distributed at the surface of nanospheres, whereas they are commonly aggregated at the corners of magnetic nanocubes.^[^ ^112^ ^]^

How cells are adapted to and are affected by mechanical cues is the fundamental challenge in mechanotransduction research. It has been proven that external mechanical stimuli have a vital effect on the fate and behaviors of cells.^[^ ^113^ ^]^ MN‐based approaches have unique advantages in this research field. First, the composition and size distributions of MNs can be designed to obtain tailored responses to different magnetic fields. Owing to the ability to precisely control the applied force and location of MNs, MN‐based approaches have become a promising tool to study the dynamics and mechanical stabilities of biomolecules and biocomplexes in vitro. The mechanical stability and lifetime of vinculin‐based intermolecular interfaces, which is critical for focal adhesions and mechanosensing, are directly quantified by Le et al. using an MN‐based single‐molecule detector assay.^[^ ^114^ ^]^ Forces at different loading rates were applied through magnetic beads to the vinculin‐based interfaces. Their calculation reveals unexpectedly high mechanical stability at these force‐bearing interfaces, indicating that they can provide enough mechanical support for mechanosensing and mechanotransduction. The function of the human facilitates chromatin transcription (FACT), which consists of two subunits, in nucleosome remodeling has also been carefully investigated by MN‐based approaches.^[^ ^115^ ^]^ Repeated stretching and relaxing measurements have been performed with MTs on nucleosomes in the presence or absence of different subunits of FACT. These findings indicated that the two subunits functioned oppositely and coordinately to fulfill the function of FACT in reorganizing the nucleosome during transcription or DNA replication. In addition, integrin ligands can be easily introduced on the surface of MNs by chemical or biological modification.^[^ ^107^ ^]^ Such modification not only greatly improves the biocompatibility of MNs but also extensively expands their application perspective. For example, a previous study by MN‐based approaches demonstrated that forces exerted on cells by magnetic beads coated with ligands led to the rapid strengthening of the cytoskeleton.^[^ ^107^ ^]^ Additionally, anti‐syndecan‐4 antibody‐modified magnetic beads were introduced in the investigation of the kindlin‐integrin‐RhoA pathway by directly exerting forces on the cell surface with MTs.^[^ ^116^ ^]^ Moreover, the local nonlinear viscoelastic responses of living cells in some force‐triggered mechanotransduction processes can be studied by modified MN‐based approaches.^[^ ^23^, ^117^ ^]^ Taking advantage of microwires with superparamagnetic properties as sensors for microrheology under a magnetic field, J.‐F. Berret developed a rotational magnetic spectroscopy assay to assess the local viscoelasticity of cytoplasm of living cells.^[^ ^117^ ^]^ The shear viscosity and elastic modulus of cell cytoplasm, which suggests that the cytoplasm should be considered as a viscoelastic liquid rather than an elastic gel, were quantified by the average rotation velocity and oscillation amplitude of magnetic microwires in cells. In addition, the use of fluorescent magnetic nanoparticle (FMNP)‐based techniques allows the directed manipulation of membrane proteins at a relatively enhanced spatiotemporal resolution.^[^ ^118^ ^]^ The development of 3D MN‐based approaches has greatly contributed to the investigation of intracellular components, such as the nucleus and organelles.^[^ ^119^ ^]^ Recently, a technology interfacing 3D magnetic twisting cytometry (MTC) with confocal fluorescence microscopy was developed to image force responses in living cells.^[^ ^120^ ^]^ Ferromagnetic beads controlled by 3D‐MTC can apply rotational shear stresses in the desired direction to cells via surface receptors as mechanical stimuli. Simultaneously, confocal fluorescence microscopy is employed to quantify structural and biochemical changes in cells that represent the responses of living cells to forces. This 3D‐MTC microscopy platform allows rapid real‐time measurement of a living cell's responses to specific external mechanical signals. Additionally, because the magnetic forces on MNs can be remotely controlled and fine‐tuned by adjusting the magnetic gradient, MN‐based approaches have been readily used as micromanipulation systems for intracellular stimulation and measurements.^[^ ^121^ ^]^ Wang et al. devised multipole MN‐based systems, which achieved both submicrometer position control and piconewton force control, to apply mechanical stimuli repeatedly on the same location of the nuclear envelope.^[^ ^122^ ^]^ It was shown that the local stiffness of the nucleus envelope exhibited a polar distribution, which was attributed to the alignment of actin filaments. Moreover, force‐induced stiffening of the nucleus envelope can be observed in their measurement. This phenomenon was highly related to the reorganization and reinforcement of the load‐bearing network underneath the NE, consisting of the structural protein lamin and intracellular stress fiber actin filaments, upon mechanical stimulation. Consequently, MN‐based approaches have been promising platforms to study the mechanotransduction of target biosystems^[^ ^123^ ^]^ and tools for medical therapy.^[^ ^124^ ^]^

## 6 Optical Traps

**Figure 6:**
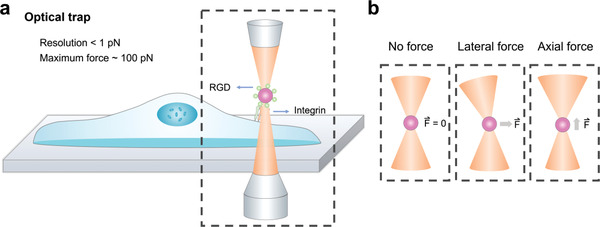
Schematic diagram of the optical trap approaches. a) A focused laser beam is applied on a dielectric bead with a micrometer diameter to provide axial or lateral forces to molecules; the dielectric beads are trapped at the center of the beam. b) Optical tweezers can trap the bead at the center of the beam. If the bead is shifted axially or laterally out of the trap center, then the diffraction of the beam can cause a restoration force on the bead, pulling it back to the center.

Optical trapping is another popular investigation approach for mechanotransduction studies.^[^ ^23d^ ^]^ OTs were first proposed and invented by Arthur Ashkin and coworker in 1970.^[^ ^125^ ^]^ The very first application of OTs in biological research was to manipulate living cells.^[^ ^126^ ^]^ However, due to the tremendous complexity of the internal structure of living cells, it is very difficult to carry out accurate quantitative mechanical measurements in vivo. The development of this application is relatively slow. Furthermore, because of the easy operation and precise measurement of extracellular space, OTs have recently been used more in in vitro experiments. Nevertheless, OTs are an effective tool for stimulating cells or specific membrane proteins mechanically, as well as quantifying mechanical cues in vivo. In a typical optical trapping experiment, a laser beam is employed to generate a light field with gradient intensity, and a dielectric particle with a higher refraction index compared to the surrounding medium is introduced to perturb cells or sense the mechano‐responses of cells^[^ ^127^ ^]^ (**Figure** **6**). Two different forces are subjected to the dielectric particle, including gradient forces and scattering forces.^[^ ^128^ ^]^ The gradient force tends to pull the particles toward the focus region of the beam along the optical direction (*z*–axis), while the scattering force pushes the particles away from the beam focus region. An efficient optical trap can be readily created when the effect of gradient forces is significantly larger than that of scattering forces. The strength of the trap relies on the refraction index and particle geometry and is influenced by the intensity gradient of the laser beam.^[^ ^127b^ ^]^ Owing to the unique penetration of light, most dielectric particles can be trapped directly inside cells and individually manipulate biological structures and interactions. Moreover, recent developments have greatly expanded its availability. This approach has also been used to investigate the dynamic behaviors of molecular motors inside living cells.^[^ ^129^ ^]^

Compared with AFM and MTs, OTs have some unique features. First, OTs can achieve high‐resolution measurements with regard to position and force and can measure displacements on the Angstrom scale and forces on the sub‐piconewtons scale.^[^ ^130^ ^]^ The temporal resolution is on the microsecond scale.^[^ ^131^ ^]^ Additionally, the use of light as a manipulation tool makes it possible to explore the mechanical properties inside cells.^[^ ^132^ ^]^ OTs can be easily equipped by optical microscopes by focusing a laser beam to create a light field with a large intensity gradient (Figure 6a). Through the interaction between the light field and the dielectric beads, the beads can be easily and stably trapped to a specific position (Figure 6b). The light gradient can be created as a 3D distribution so that both repulsive and attractive forces can be applied on the beads.^[^ ^133^ ^]^ Polystyrene and silica microspheres are the most common beads in OTs. Furthermore, similar to the design of MTs, trapped beads are used as handles to manipulate molecules or cells (Figure 6a). The position of the beads can be used to probe the movements, and the force exerted on the molecule or cell can be calculated simultaneously. Moreover, different chemical or biological surface modifications can functionalize the beads to achieve different goals. For example, optical traps can be used to manipulate nanobeads with ligand‐modified surfaces to interact with cells. A study using submicron‐diameter beads with an optical trap showed that high levels of passive rigidity of matrix molecules were needed to induce strong linkages between the ECM and the cytoskeleton.^[^ ^134^ ^]^ However, a fundamental technical concern is that the high intensity at the focus of the trapping laser beam can result in significant local heating, hence causing cell photodamage^[^ ^135^ ^]^ and potential impact of the observation results.^[^ ^136^ ^]^ The local heating effect, which can influence the enzymatic activities and alter the local viscosity of the microenvironment, is systematically calculated and analyzed by Peterman et al.^[^ ^135a^ ^]^ In their study, the values for the laser‐induced heating of varied dielectric microparticles in different media were experimentally determined and summarized. Near‐infrared lasers are employed to reduce the photodamaging effect.^[^ ^137^ ^]^ Alternatively, using two divergent laser beams rather than a single focused beam and employing heavy water as the medium rather than usual water can both weaken this effect.^[^ ^138^ ^]^

The common application of OTs is to apply mechanical stimuli to the outer cell membrane and characterize the response, either to exert forces on specific membrane proteins or to measure the mechanical parameters of cells. Before an optical trapping experiment, polystyrene beads covered by target ligands are selected. The size of the beads depends on the membrane to be investigated. The basic configurations of OTs include a position detector based on a quadrant detector with high spatial and temporal resolution. The first step before the measurement is calibration. In this process, dielectric beads are trapped in a buffer far from the cell, and the thermal fluctuation of the beads is introduced to calculate both the trap stiffness and the conversion factor of the position detector. Although the basic function of OTs is manipulating cells from the outer membrane, direct application of mechanical stimuli and force measurement inside living cells are unique features of OTs. The dielectric beads used in OTs are small enough (micrometer) to enter cells or organelles by phagocytosis. Meanwhile, the beads can be manipulated by a laser as force probes directly. However, in this case, trap calibration can be a great challenge. The components inside cells usually show viscoelastic properties, and the interaction between dielectric beads and the cytoskeleton often contributes greatly to the parameters. In addition, since the trap stiffness and the conversion factor vary largely with the size of the beads in the micrometer range,^[^ ^23d^ ^]^ precise calibration inside cells with beads of unknown size becomes extremely difficult. To overcome this obstacle, Fischer and coworkers designed a general calibration approach to obtain the trap stiffness and the conversion factor for dielectric beads of unknown size in a medium with unknown viscoelastic properties.^[^ ^139^ ^]^ Following this concept, these researchers proved its availability in a solution of entangled F‐actin and compared it within a simple viscous medium.^[^ ^140^ ^]^ A combination of thermal fluctuation and forced oscillation was employed in this approach to quantify both trap calibration parameters and viscoelastic properties of the medium. Because thermal fluctuation is a kind of passive recording and forced oscillations are considered active recordings, this approach is also known as active‐passive calibration.^[^ ^23^, ^139^ ^]^

OTs are extremely suitable for measuring the molecular bond strength involved in mechanotransduction both in vitro and in vivo. As reviewed by Bustamante et al.,^[^ ^141^ ^]^ different setups of OTs, including standard OTs, fleezers (dual‐trap OTs with a confocal microscope),^[^ ^142^ ^]^ and angular OTs,^[^ ^143^ ^]^ have provided powerful support for the investigation of single‐molecule biophysics. Many specific cell membrane receptors form adhesion complexes with extracellular components or neighboring cells. Quantitative investigation of how their binding kinetics are regulated by mechanical cues can contribute greatly to the understanding of the underlying principles. Optical trapping approaches yield direct information about the lifetime and association/dissociation constant of individual receptor‐ligand molecular pairs. To achieve this mission, nanobeads functionalized with different ligands are employed to mechanically manipulate molecular pairs^[^ ^144^ ^]^ (Figure 6a). Typically, the strength of the bond can be described as the rupture force at a specific loading rate.^[^ ^145^ ^]^ Based on the Bell‐Evans model,^[^ ^146^ ^]^ the dissociation rate at zero force can be calculated. The experiment carried out by Sako et al. on the strength of cadherin bonds to the cytoskeleton regulated by catenin using OTs is pioneering work.^[^ ^147^ ^]^ The bond between integrin and fibronectin, a fundamental complex for cell adhesion, has also been successfully characterized by OTs.^[^ ^148^ ^]^ Additionally, optical trapping methods have functioned effectively in other aspects of mechanotransduction research, such as quantifying active forces and movements on cell surfaces^[^ ^134^, ^149^ ^]^ or inside cells^[^ ^126^, ^150^ ^]^ and measuring the mechanical properties of different cells.^[^ ^136^, ^151^ ^]^ A noninvasive experimental scenario combining optical trap and light‐sheet microscopy developed by Kapil Bambardekar enabled the ability to directly probe the mechanical properties of epithelial cell junctions in *Drosophila* embryos.^[^ ^152^ ^]^ The subcellular cell‐cell junction is precisely trapped and manipulated without a transparent particle. Furthermore, pull‐and‐release experiments are achieved efficiently. Their results revealed the magnitude of the tension at cell junctions and that a simple viscoelastic model can basically describe the time‐dependent properties of the junction mechanics. Recently, OTs have also been combined with other instruments, such as fluorescence microscopy and microfluidic platforms, to simultaneously detect multiple signals.^[^ ^153^ ^]^ In the cross‐instrument approach developed by Vasse et al., a dual‐beam optical trap is employed to capture an isolated single macrophage, hence greatly reducing the effect caused by cell‐cell interactions and surface adhesion.^[^ ^153a^ ^]^ Their strategy allows the detection and tracking of the response of a cell to single biomechanical or biochemical stimuli at high spatiotemporal resolution in real time. Also, optical traps offer a powerful platform for single molecular‐ or single‐cell‐level microrheology measurements and have been successfully exploited for a variety of applications.^[^ ^154^ ^]^ Nishizawa et al. devised a feedback‐tracking microrheology approach to track particle probes stably inside fibroblasts and epithelial‐like HeLa cells with constant cytoplasmic perturbations. They also developed a complex theory combining the fluctuation‐dissipation theorem (FDT) into positional feedback to understand the data, which reveals the glassy dynamics of the cytosol.^[^ ^154a^ ^]^ Similarly, both extracellular and intracellular viscoelasticity of different types of ECMs and cells were measured by Staunton et al. using optical trap‐based microrheology to assess the ability of cells to remodel and adapt to their microenvironment.^[^ ^154b^ ^]^ Their work demonstrated that compared with normal cells, tumor cells commonly possess higher mechanical plasticity to adapt to multiple environments. Because of the dramatic complexity of the brain and inherent difficulties in manipulating cells and molecules located deep within tissues, it is difficult to directly investigate mechanotransduction in neural systems. With recent advances in OTs and optical imaging devices, OTs have fundamentally overcome the main challenges and hence have become an established tool in neuroscience.^[^ ^155^ ^]^

## 7 MFPs

Recently, a visualization approach based on force‐sensitive molecules has drawn great attention in the investigation of mechanotransduction.^[^ ^23^, ^156^ ^]^ This technique is also named MFPs or molecular force sensors (MFSs). Through molecular‐based design, MFPs have overcome some of the limitations of traditional methods. The throughput of MFPs is relatively high because a large number of probe molecules can be visualized at one time by fluorescence imaging. Meanwhile, owing to that each probe molecule detects mechanical information on the piconewton scale for individual interactions, MFPs can also reach single‐molecule resolution.^[^ ^157^ ^]^ The combination of high throughput and high‐force resolution makes MFPs a super powerful tool for quantitively investigating adhesion and mechanotransduction in different biological systems, including living cells and even whole organisms.^[^ ^158^ ^]^

**Figure 7:**
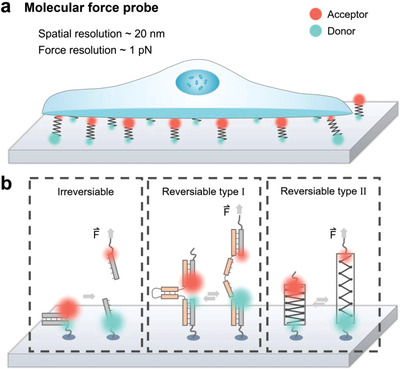
Schematic diagram of the molecular force probe (MFP) approaches. a) The basic components of the MFPs. Typically, a single MFP consists of a fluorescence donor‐acceptor pair and a force‐sensitive element. The force‐sensitive element responds to probe forces, and the fluorescence pairs report the signals. b) Categories of the MFPs. Based on the force‐sensitive elements used in the system, MFPs can be categorized into three different classes: irreversible sensors (left), type I reversible sensors (middle), and type II reversible sensors (right). The irreversible sensors can only be used once. Depending on the signal output results, reversible sensors can be divided into two types. Type I reversible sensors output discrete signals, and there is usually a threshold force that is defined as the transition point. Type II reversible sensors output continuous signals.

Since Albrecht and coworkers successfully completed the first MFP‐based experiment approximately two decades ago, although the basic design principle still adopts force‐to‐fluorescence conversion to visualize force information, MFPs have developed considerably.^[^ ^159^ ^]^ Typically, the basic components of an MFP system include a force‐sensing element and a fluorescence reporter.^[^ ^157a^ ^]^ Fluorescence reporters are responsible for reporting mechanical cues, including tension force and displacement by fluorescence resonance energy transfer (FRET),^[^ ^149^, ^160^ ^]^ fluorescence quenching,^[^ ^161^ ^]^ nano‐surface energy transfer,^[^ ^161a^ ^]^ or fluorescent labeling of force‐sensitive probes.^[^ ^162^ ^]^ The donor‐acceptor pairs based on FRET are the most common fluorescence reporters in MFPs (**Figure** **7**a). Depending on the molecules used for force sensing, the spatial and force resolution can reach 20 nm and 1 pN, respectively.^[^ ^157a^ ^]^ In the traditional design of MFPs, one end of the probe is immobilized to a confined surface by chemical modification or physical interactions, and the other end is often functionalized with ligands to bind the target receptors on the membrane of cells.^[^ ^157^, ^161^ ^]^

For the force‐sensing element, a switch‐like molecule with well‐defined structures, such as proteins and DNA duplexes or molecules with entropic spring‐like properties and random coil conformations, such as elastin‐like proteins or polypeptides (ELPs) and polyethylene glycol (PEG), can both function effectively. There are many categorization methods to divide MFPs into different classes. For example, they can be categorized by synthesis methods, differences in components, construction materials, and signal output approaches.^[^ ^23^, ^157^, ^163^ ^]^ Here, we adopt a classification scheme that has been recently employed by Salaita's group and Li's group to categorize MFPs.^[^ ^23^, ^164^ ^]^ This scheme is based on the sensing mechanisms of MFPs and categorizes them into three different classes: irreversible, reversible type I, and reversible type II (Figure 7b). In this review, we briefly introduce the advantages and drawbacks of the different types. Detailed information can be found in recent reviews.^[^ ^23^, ^164^ ^]^

The class of MFPs consisting of irreversible force‐sensing elements is usually characterized by irreversible interactions or structural transitions, such as the irreversible breakage of chemical bonds and the dissociation of complementary DNA strands. Consequently, the irreversible force probe can only be used once and will subsequently lose its force‐sensing capability. Irreversible MFPs often have a two‐state energy landscape, that is, a bound state and an unbound state. There is an energetic barrier between the two states, and the barrier can only be crossed once from the bound state to the unbound state. Therefore, the signals output by irreversible force probes always exhibit digitality and represent energetic barrier crossing events. The type I reversible force probe is also known as the reversible digital force probe. Similar to the irreversible force probe, a two‐state system including a folded state and an unfolded state is employed. Herein, a digital‐like discreteness output signal is produced by this type of MFP. However, the transition between the two states is a reversible process and is typically force dependent. The force‐sensing element usually stays in the folded state at low force but stays in the unfolded state at high force. To measure forces precisely, the kinetics and mechanical properties of the MFS should be well characterized. DNA hairpins and titin are the most employed molecules.^[^ ^161^, ^165^ ^]^ They can provide reliable two‐state transitions. The transition is around a threshold force. Only forces higher than the threshold force can be reported. This threshold value can be fine‐tuned within a range by varying the DNA sequences and changing different proteins. Meanwhile, the fraction of opened probes can be quantified proportionally to the fluorescence intensity by employing standard calibration methods.^[^ ^164^, ^166^ ^]^ However, a problem that cannot be ignored is that the threshold force between the two transition states is usually loading rate dependent. The transitions in different directions (folding or unfolding) may occur under different forces, resulting in hysteresis at relatively high force loading rates.^[^ ^167^ ^]^ Hence, guaranteeing that the loading rate used does not cause force hysteresis is vital for the success of the measurement. Type II reversible probes are quite different from the two classes introduced before. Type II reversible probes are also called reversible analog force probes. The output signals of these probes are usually continuous and exhibited as a proportional function of the magnitude of applied force within a dynamic range.^[^ ^160b^ ^]^ Elastomeric polymers and proteins are often employed to achieve fast and hysteresis‐free reversibility.^[^ ^168^ ^]^ Classic PEG‐based MFPs are the most successful examples.^[^ ^160b^ ^]^ When an external force is applied to these elastomeric polymers, their conformations will instantly deform and reach equilibrium. After removal of the force, these polymers will recover to their zero‐force conformation. By optimizing the structures and components of the elastomeric polymers, the maximum tunable force‐sensing range of this MFP can extend from 1 to 25 pN.^[^ ^163^, ^169^ ^]^ When investigating continuously changing forces in specific mechanotransduction processes, reversible analog force probes have unique advantages. However, it is difficult for them to function effectively when estimating single ligand‐receptor forces.^[^ ^164^ ^]^

Owing to their designability and programmable characteristics, MFP‐based approaches have become a powerful tool to probe and measure not only extracellular adhesive forces but also intracellular mechanotransduction processes. DNA‐based MFPs, traditionally including double‐strain DNA and DNA hairpins, are one of the most popular methods currently. Recently, a digital force probe based on a DNA hairpin with tunable force response thresholds, functionalized ligands, and signal reporters was employed to quantitatively image the integrin force profile in early cell adhesion.^[^ ^165a^ ^]^ Similarly, in the work reported by Liu et al., the mechanism of force and receptor‐triggered T‐cell activation was carefully investigated by DNA hairpin‐based gold nanoparticle tension sensors.^[^ ^165c^ ^]^ To overcome the drawback of DNA‐based MFPs, in which it is rarely possible to measure ligand‐receptor interactions in a higher force regime (>20 pN), Li et al. developed a reversible shearing DNA‐based MFP that can achieve a high measurable force of 60 pN with force resolution to the piconewton scale.^[^ ^170^ ^]^ Reversibly probing relatively high forces generated by ligands without destabilizing or disrupting the adhesion site empowers these reversible shearing DNA‐based MFPs to directly reveal the differences between load‐bearing integrins. Besides the improvement in the maximum measurable force and force resolution, the temporal resolution of DNA‐based MFPs has also been dramatically advanced. Adam B. Yasunaga and Isaac T. S. Li devised a footprint assay derived from nonequilibrium DNA‐based MFPs to probe and picture the rapid adhesion events in rolling adhesion processes.^[^ ^171^ ^]^ This tool successfully quantified the adhesion force distribution with a dynamic force range from 0 to 18 pN and revealed the underlying mechanism of rolling adhesion. In addition, DNA origami has also been introduced to design and program multivalent tension MFPs to investigate cellular traction forces.^[^ ^172^ ^]^ Due to the unique ability to characterize interactions between ligands and receptors that are widely distributed on cell membranes, DNA‐based MFPs have been used to topologically and stoichiometrically sort and classify different cells.^[^ ^173^ ^]^ Despite a lack of fundamental understanding and precise characterization, there have also been numerous technological advancements in biosensing and related applications.^[^ ^174^ ^]^ On the other hand, with the development of molecular biology, MFP‐based approaches have been exploited to promote cell mechanotransduction and mechanically mediate the cell fate of stem cells.^[^ ^175^ ^]^ Furthermore, a well‐designed optical sensor based on MFPs has been successfully employed to study abnormal nuclease spatial dynamics at the subcellular level on cancer cell membranes.^[^ ^176^ ^]^ To conclude, despite the inevitable challenges, MFP‐based approaches provide new insight from the molecular view into how mechanical cues transduce and regulate cell fate.

## 8 Micropipette aspiration

The precise manipulation of single cells with increasing complexity at the microscale is a fundamental challenge in the investigation of mechanotransduction. Another well‐known tool to accomplish this mission is MPA. The first MPA‐based experiments were performed and analyzed by Evans in 1973. The mechanical properties of red blood cells were studied by micropipette deformation.^[^ ^177^ ^]^ With several decades of sustained improvement and application, micropipettes have been utilized in a wide range of investigations in the field of cell mechanics, including adherent cells^[^ ^178^ ^]^ and suspended cells.^[^ ^177^, ^179^ ^]^ For example, MPA was recently employed to investigate cell nucleus deformation in response to mechanical stresses by applying forces to nuclei within intact cells, thus establishing a unified linear viscoelastic model that revealed that not only lamin A but also lamin B1 contributions to nuclear stiffness.^[^ ^180^ ^]^

**Figure 8:**
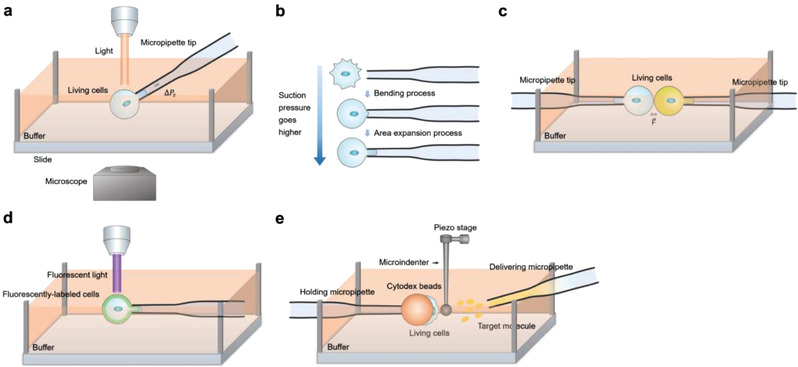
Schematic diagram of the micropipette aspiration (MPA) approaches. a) The basic setup of MPA experiments. The forces applied to living cells can be produced by differential pressure. The suction pressure is generated by a pump or an adjustable water reservoir that is connected to the micropipette. The changes in the cells were recorded by a microscope and analyzed by imaging processing methods. b) Schematic diagram of the bending process and area expansion process. c) MPA approaches can be used to determine the intracellular forces. A dual‐pipette aspiration setup is introduced in such experiments. d) Confocal microscopy and fluorescent labeling methods can be combined with MPA approaches to quantify the related protein expression and cell mechanotransduction by calculating the fluorescence intensity. e) The sensing and response of living cells can be determined by the MPA approaches. Microindentation techniques are introduced to locally deform an attached cell and measure the mechanical properties of the cell. An additional micropipette is introduced to deliver some stimulating molecules.

In a typical MPA experiment, the cell shape change during aspiration processes was imaged and analyzed (**Figure** **8**a). A pump or an adjustable water reservoir is employed to generate suction pressure. The suction pressure can be calculated by the height difference between the top of the reservoir and the tip of the micropipette.^[^ ^181^ ^]^ Depending on the types and modulus of the cells and components to be investigated, the magnitude of the suction pressure can be adjusted from ≈1 Pa to several kPa.^[^ ^182^ ^]^ The temperature of the chamber and the position of the micropipette tip are controlled precisely by a micromanipulator. Generally, the diameter of the micropipette is known and often much smaller than that of the cells under investigation. After the suction pressure is exerted, the cell will be partially or totally pulled into the micropipette. A microscopy system including an inverted microscope and a CCD camera is usually used to monitor and track cell changes. The cell changes are illustrated as a sequence of images. As reported by many studies, with increasing suction pressure, the cell demonstrated distinct bending and expansion processes^[^ ^183^ ^]^ (Figure 8b). The membrane shows incompressible properties during the bending process. However, during the area expansion process, due to the large expansion of the surface area, the membrane is strained.^[^ ^183^ ^]^ Although the basic hardware requirements are relatively simple, the actual experimental process is much more complicated. A flow can cause great effects on the calculation of the suction pressure. The pressure drop along the micropipette should be considered carefully. For the detailed analysis process, readers can refer to ref. [181]. Moreover, the adhesion between cells and the glass surface is a common obstacle in MPA experiments.^[^ ^184^ ^]^ This can lead to overestimation of the cell's mechanical properties. To overcome this obstacle, several different well‐designed protocols have been devised.^[^ ^185^ ^]^

Biomechanical models are critical for the accurate derivation of cellular mechanical properties based on the structural changes in cells from MPA experiments. Theret et al. proposed a simple model to quantify the moduli of the samples.^[^ ^186^ ^]^ In this model, the samples are assumed to be incompressible and show homogeneous linear‐elastic properties. However, real living cells are much more complex than assumed by the model. To calculate the mechanical parameters of living cells more accurately, a series of efficient models have been devised. Depending on the assumptions made, these models can be roughly divided into two different types. The first type is a continuum model, which is similar to the model used by Theret et al.^[^ ^186^ ^]^ and assumes that the cells exhibit homogeneous properties. Plaza et al. developed a model to address the effect of Poisson's ratio.^[^ ^179b^ ^]^ If the cells are suspended and nearly spherical, then the finite size should be taken into account for precise calculation. Zhou et al. extended the standard model into the standard neo‐Hookean solid model to carefully investigate the influence of the micropipette radius.^[^ ^187^ ^]^ Moreover, a parameter related to the relative size of the cells was employed by Plaza et al.^[^ ^188^ ^]^ The value is based on the ratio between the micropipette radius and the radius of cells. The models discussed above do not consider the time‐independent mechanical properties. To address the viscoelastic properties of living cells, the creep function, which includes time as a variable, should be considered in the model. Based on this principle, time‐dependent models that are widely used in practice have been set up in different ways.^[^ ^178^, ^189^ ^]^ As the suction pressure increases, the deformations of the cells become larger. The mechanical behavior of the cells changes from solid‐like to fluid‐like.^[^ ^190^ ^]^ By modifying the solid‐like models, the apparent viscosity of the living cells can be quantified.^[^ ^178^, ^179^, ^185^ ^]^ Although models based on the assumption of homogenous mechanical properties are extensively useful in quantitative analysis, mechanical properties are usually distributed heterogeneously and anisotropically in living cells. Therefore, the second type of model that considers the effect of the microstructures and subcellular components was derived. Depending on the assumption of the cell structures, these models mainly include diffusion models,^[^ ^178c^ ^]^ poroelastic models,^[^ ^191^ ^]^ liquid and cortex models,^[^ ^189^, ^192^ ^]^ and actomyosin cortex models.^[^ ^76^, ^193^ ^]^ Due to the tremendous complexity of intracellular structures and molecules, biomechanical models cannot fully describe every detail of the mechanical properties of living cells. Developing more suitable models that can be applied under different conditions is an active area in the research of MPA‐based approaches. For instance, the discrete particle model developed by Lykov et al. successfully described the changes in the cell membrane and cytoskeleton during the aspiration regime.^[^ ^194^ ^]^ To find the detailed information in this direction, readers can refer to ref. [181]. In addition to developments in theoretical models, investigating cellular processes outside of cells to decipher the complexity step by step has also been a widely used and powerful method to capture the details of mechanotransduction. To study membrane homeostasis and intracellular transport, Sorre et al. performed a combination measurement including force and fluorescence tests on the membrane by a developed MPA‐based experimental approach.^[^ ^195^ ^]^ Their results revealed that curvature‐triggered lipid sorting was mediated by a mechanism combining the collective behavior of lipids and the amplification effect of lipid‐clustering proteins. Similarly, this deciphering strategy was adopted to scrutinize cellular functions by investigating the mechanical behaviors of giant unilamellar vesicle (GUV) model systems and force‐induced nanotubes.^[^ ^196^ ^]^

In addition to the development and evolution of biomechanical models, MPA has been combined with other techniques to carry out more complex measurements. Intercellular interactions usually exhibit dominant effects in controlling morphogenesis. To probe the mechanics and dynamics of cell‐cell contact, especially their adhesion strength, a dual‐pipette aspiration (DPA) system has been devised.^[^ ^197^ ^]^ The setup of the DPA system is also relatively straightforward. Two antiparallel micropipettes were employed to precisely manipulate cells at the microscopic scale (Figure 8c). As a result, more than two cells can be manipulated simultaneously by the DPA system.^[^ ^197^, ^198^ ^]^ The MPA approaches thus show great potential in investigations of cell growth and aggregation.^[^ ^3^, ^199^ ^]^ Recently, the combination of MPA and fluorescent labeling methods with confocal microscopy (Figure 8d) has been employed to not only quantify the global and local mechanical properties of living cells but also study the molecular mechanisms of cellular mechanotransduction.^[^ ^200^ ^]^ The classic work by Robinson et al. successfully deciphered cortical mechanotransduction from molecular to cellular scales by combining MPA approaches and fluorescent labeling techniques.^[^ ^200a^ ^]^ Their analysis indicated that myosin II shared forces with different actin crosslinkers together in cortical mechanotransduction processes. Moreover, myosin can cause potentiating or inhibitory effects on certain actin crosslinkers.^[^ ^200a^ ^]^ Additionally, a single‐cell biological force determination assay developed by González‐Bermúdez et al. combining MPA and 3D confocal analysis was applied to mouse CD4^+^ T‐cells to assess the deformability and internal ordering relationship inside cells.^[^ ^201^ ^]^ Their results reveal that the size of the nucleus is the most effective factor influencing the overall deformability of cells compared to other cytoskeletal or geometrical features. In localized analysis, the mechanosensing and mechanotransduction of membrane‐embedded ion channels and proteins have been investigated with the help of the combination of MPA and single‐particle tracking. In addition to molecular observations, microindentation techniques have also been introduced to carry out localized analysis together with MPA.^[^ ^200b^ ^]^ The basic setup includes a profile microindenter and a holding micropipette to grab cells (Figure 8e). This system enables the visual observation of adherent cells in profile while measuring their mechanical properties and applying mechanical stimuli simultaneously. Also, soluble factors and stimulating molecules can be added into the system by an additional delivery micropipette.

Thanks to the relatively simple setup and ease of operation, MPA has been a highly versatile technique and has adapted to many experimental systems to study cell mechanics, mechanotransduction, and cell dynamics spanning different spatial resolutions.^[^ ^180^, ^202^ ^]^ However, the relatively low throughput and force resolution are inevitable limitations of MPA. Typically, the throughput of MPA is ≈20 cells per hour.^[^ ^181^ ^]^ The forces it can apply are extremely difficult to reach the piconewton level, which has been achieved by many other techniques, such as AFM. Furthermore, because the size and geometry of glass micropipettes are difficult to precisely control, the accuracy and sensitivity of the measurement can be greatly affected. Fortunately, with the recent increasing development of microfluidic and optical techniques, the application of MPA has been largely expanded.^[^ ^202^, ^203^ ^]^ For instance, the throughput of hydrodynamic micropipettes that combine microfluidic devices and MPA can reach an exceptionally high throughput level of ≈1000 cells per hour.^[^ ^202^, ^204^ ^]^ Moreover, for novel optical interferometry‐based MPA, subnanometer spatial and real‐time temporal resolution has been attained, thanks to a unique data acquisition method using the phase variations in backscattered light from cell surfaces.^[^ ^205^ ^]^ Also, a combination setup of MPA and phase‐modulated surface acoustic wave microfluidic devices has been developed to measure cellular compressibility as well as Young's modulus simultaneously to evaluate cell elastic properties.^[^ ^206^ ^]^ Besides the in vitro measurement, a technique named micropipette force sensor (MFS) based on MPA has achieved precise force detection in vivo on single cells and multicellular microorganisms with a force resolution as low as 10 pN.^[^ ^207^ ^]^

## 9 Discussion and Perspective

Mechanosensing and mechanotransduction of cells play an important role in many diseases, such as cardiovascular diseases and cancers. How cells respond to mechanical cues over different time scales may become the key to understanding the development of these diseases. In this article, we briefly review the recently developed approaches for investigating the mechanical properties and mechanotransduction of biological systems crossing different force and length ranges. These platforms provide unique insights to interpret how cells communicate with their environment mechanically. The unique characteristics of these approaches are summarized in **Table** **2**. Each approach has advantages and limitations. Therefore, selecting a suitable method according to the system of interest is vital for the success of the experiment.

**Table 2:** Overview of the primary characteristics of commonly used approaches for measuring biological forces

Approaches	Microbead‐based TFM^[^ ^22^, ^32^, ^87^ ^]^	Micropillar‐based TFM^[^ ^22^, ^53^, ^87^ ^]^	Atomic force microscopy (AFM)^[^ ^17^, ^23^, ^132^, ^183^ ^]^	Magnetic tweezers (MTs)^[^ ^22^, ^23^, ^132^, ^183^ ^]^	Optical tweezers (OTs)^[^ ^22^, ^23^, ^132^, ^183^ ^]^	Molecular force probes (MFPs)^[^ ^23^, ^87^, ^168^ ^]^	Micropipette aspiration (MPA)^[^ ^17^, ^181^, ^183^ ^]^
Dimension	Microscale	Microscale	Microscale, nanoscale	Microscale, nanoscale	Microscale, nanoscale	Microscale, nanoscale	Macroscale or microscale
Concept	Reconstruct cell‐generated traction forces by computational analysis of the direction and the magnitude of elastic substrate deformation	Directly measure the local traction by the bending of individual pillar	Direct measurement of forces and displacement by nanoindentation and calculation of mechanical properties by biomechanical models	Manipulate magnetic beads by magnetic field to apply force on cells or molecules in vitro or in vivo	Manipulate dielectric beads by light field to apply force on cells or molecules in vitro or in vivo	Convert force signal into fluorescence signal by the changes of force‐sensing molecules	Using biomechanical models to establish the relationship between the suction pressure and the aspirated profiles of the sample
Spatial resolution [nm]	10^3^	<1	0.5–1	1–10	0.1–2	≈20	25
Force resolution [pN]	≈10^3^	≈10	≈1	<1	<1	≈1	N/A
Displacement range [nm]	10^3^–3 × 10^3^	N/A	0.5–10^4^	5–10^4^	0.1–10^5^	10–10^3^	N/A
Force range [pN]	10^3^–10^5^	50–10^5^	10–10^4^	10^−3^–10^2^	0.1–100	1–100	0.3 (0.1–10 mbar)
Probe size [µm]	N/A	>1	≈10 nm for sharp tips ≈10 µm for beads	0.5–5	0.25–5	Molecular scale	N/A
Advantages	Easy to set up (using standard lab equipment and fluorescence microscopy)	Easy to tune the substrate rigidity depending on the cells; direct measurement	High loading rate range; high throughput; Large displacement; easy to modify specific molecules on the surface of the probe	Remote control; almost no damage to living samples; specific interactions	High resolution in both position and force measurement	High resolution with standard fluorescence microscopy	Simple to set up; easy to operate
Limitations	Low spatial and force resolution; heavy computational overhead for the reconstruction process	Forces are independent for posts Fabrication	High noise; large minimal force; difficult to operate inside cells.	Force hysteresis	Difficult to operate; nonspecific; sample damage by laser heating	Long sample preparation time; difficult to reflect the real cellular situation; limit to 2D	Difficult to control accuracy; Low throughput; low force resolution.

However, we should be aware that even the measurements were performed on the same system under a similar environment and basic assumptions about materials were kept consistent, the results calculated from different technologies may still differ from each other, as shown in a recent publication by Wu et al.^[^ ^208^ ^]^ In their study, a comparison of different approaches was performed by using identical cell preparations and cell culture medium provided by the same source. The stiffness, represented by Young's modulus, of MCF‐7 breast cancer cells ranged from tens of Pa quantified by OTs; however, the particle tracking microrheology of suspended cells was ≈1 kPa by AFM with dull probes but ≈10 kPa with sharp AFM probes in parallel plate rheometry. Such a wide distribution of mechanical properties suggested the fundamental effect of the force profile on the mechanical sensing and response of cells. Here, we briefly discuss the technical reasons that resulted in these differences. For detailed analysis and experimental information, readers can refer to ref. [208]. The reason why these discrepancies appear between different approaches mainly lies in several different aspects. Issues of the heterogeneous structure of cells and the noncontinuous nature of the cytoskeleton result in the asymmetric spatial distribution of cell mechanical properties.^[^ ^209^ ^]^ The nuclear region is typically stiffer than the cell periphery;^[^ ^210^ ^]^ hence, the tested location of the probe on cell surfaces can cause a great effect. Then, the profile of probe‐cell contact is also important. Among the moduli yielded by AFM‐based measurements, the larger probe typically produced a substantially lower value^[^ ^208^ ^]^ for its lower sensitivity to the local mechanical properties of cells. In addition, the increased prestress level caused by sharp conical probes compared to the dull probes in the AFM‐based experiments can be used to explain this phenomenon, similar to the observations over other viscoelastic polymer solutions and soft materials.^[^ ^211^ ^]^ Furthermore, direct force exertion on cells, such as AFM, or applying forces by molecular links between the cell surface and functionalized probes, such as OTs and MTs, can cause profound influences on the measured values. Besides, the timescale factor and loading rate can take great responsibility for the differences in the results obtained by different approaches. In AFM‐based measurements, higher indentation speeds frequently resulted in larger elastic moduli,^[^ ^212^ ^]^ while the regulation rules of some other mechanical parameters of cells by loading rates were ambiguous.^[^ ^208^, ^210^ ^]^ It is worth noting that the cellular structure is the determining factor for its mechanical properties, which are generally dynamic rather than steady. For example, the measurement of internal stresses or tensions can be altered by molecular motors and fibronectin, as well as the concentration and crosslinking density of the cytoskeleton, which is normally considered the dominant factor in cell stiffness.^[^ ^209^ ^]^ Therefore, the modulus obtained by applying forces that persist for minutes can vary dramatically compared with that obtained with a quick measure on a subsecond timescale. Finally, disparities can be introduced into the system with different analytical methods for the primary raw data. It absolutely matters which assumption is adopted in the analytical models, such as the linear elasticity for AFM‐based measurements and viscoelastic behaviors for OTs and MTs. For example, the conversion factor and Poisson's ratio are important for determining the cell stiffness. Typically, the value of Poisson's ratio ranges from 0.3–0.5. However, the real situation must be complex because cells should be literally considered poroelastic materials.^[^ ^76b^ ^]^ In conclusion, delicately designing the experimental procedures (e.g., indentation depth and loading rates), defining the measuring location of the cells (e.g., nuclei region or periphery region), selecting suitable probes (e.g., geometry and size), and adopting appropriate models (e.g., based on experimental context and assumptions) can make the obtained parameters valid to withstand the trial.

Besides the continuous development of the techniques for more accurate measurement of the mechanical properties of cells and tissues, it is highly demanding to expand these tools for measuring mechanical properties and forces at the subcellular level. The mechanosensing that occurs on cell plasma membranes, the mechanical response of the cytoskeleton and intracellular organelles contributes greatly to the active mechanisms and pathways of mechanotransduction.^[^ ^213^ ^]^ For example, the cell nucleus, the largest and stiffest organelle, which is tightly integrated into the structural network of the cell linker complexes of the nucleoskeleton and cytoskeleton (LINC), has been implicated in multiple mechanotransduction processes.^[^ ^214^ ^]^ Current opinions hold that extracellular mechanical stimuli generated from the ECM or surrounding neighboring cells can modulate NE composition and further regulate nuclear morphology and chromatin organization.^[^ ^215^ ^]^ In addition, it has recently emerged that the mechanical properties of the cell nucleus directly play a crucial role in mechanosensing during cell migration in 3D environments, especially transiting through a narrow constricted space that is smaller than the nuclear size.^[^ ^16^, ^216^ ^]^ A study based on MTs has successfully applied forces on isolated nuclei, hence revealing that nuclei are able to directly respond to forces by tuning their stiffness to resist mechanical stress.^[^ ^217^ ^]^ Another signal transduction mechanism involving the nucleus and NE is the nucleocytoplasmic permeability barrier, which has been recently investigated and proven to be an essential component of intracellular mechanotransduction processes.^[^ ^218^ ^]^ Accordingly, more efforts are urgently needed to focus on studies of intracellular mechanobiology. Although several technologies are capable of performing force measurements inside cells, novel setups and devices or new combinations of different technologies need to be devised and developed to inquire deeply and profoundly into the underlying mechanisms.

Another technical challenge is to measure the interplay between mechanical and chemical signals. A wide variety of protein expression levels are regulated by mechanical signals, but how this network works has yet to be explored. It remains challenging to simultaneously measure the mechanical and chemical signals. Combining force and fluorescence measurements has recently been proven to be an efficient way to directly probe force‐induced biochemical signaling.

In addition, most methods thus far have only limited temporal resolution, and probing dynamic mechanical changes remains difficult; it requires that the force‐induced changes on the probes are reversible and the data acquisition and processing speed is sufficiently fast. Another challenge is to study the long‐term mechanical effect (e.g., cell division and differentiation). It requires the force measurement system to be stable over a long period of time (e.g., a few days). However, most current approaches can have considerable drift due to the mechanical design of the instrument or the change in the environmental conditions. In some methods, anti‐drift feedback systems have been introduced, which opens up new possibilities to study mechanical effects on the timescale of days.^[^ ^219^ ^]^ We believe that with the continuous improvement of the stability and resolution of these approaches, as well as the development of new techniques,^[^ ^220^ ^]^ mechanobiology can be studied in more detail.

## Conflict of Interest

The authors declare no conflict of interest.
